# Eighth International Symposium on Recent Advances in Environmental Health Research

**DOI:** 10.3390/ijerph9051626

**Published:** 2012-05-04

**Authors:** Paul B. Tchounwou

**Affiliations:** National Institutes of Health RCMI - Center for Environmental Health, College of Science, Engineering and Technology, Jackson State University, 1400 Lynch Street, Box 18750, Jackson, MS 39217, USA; Email: paul.b.tchounwou@jsums.edu; Tel.: +1-601-979-2153; Fax: +1-601-979-0570

This special issue of International Journal of Environmental Research and Public Health is dedicated to the publication of selected papers presented at the Eighth International Symposium on Recent Advances in Environmental Health Research. The Symposium was organized by Jackson State University (JSU) from September 18-21, 2011 at the Marriott Hotel in Jackson, Mississippi. It was built upon the overwhelming success of seven previous symposia hosted by JSU.

The program of the *Eighth International Symposium on Recent Advances in Environmental Health Research* provided a strong forum to discover the latest scientific advances in the areas of environmental science and public health. The Symposium’s program had continued to have a special appeal to scientists and engineers who have been committed to bioenvironmental and public health research; studying the toxic mechanisms of action of various environmental agents, developing new approaches for detecting or remedying environmental damage, identifying and characterizing genes involved in the manifestation of environmentally-related diseases, conducting basic and translational research, and providing the public and policy makers with scientific tools that are critical for environmental and human health decision-making [1]. Hence, the symposium was of special interest to toxicologists, environmental chemists and biologists, epidemiologists, public health officials, and civil and environmental engineers interested in environmental and public health research and education. Also, the symposium series continued to offer unparalleled opportunities for networking and exchange of ideas, leading to scientific collaborations, resources sharing, and strategic planning for multi- and inter-disciplinary approaches to environmental and public health research [2].

Building on the foundation of the first (2004), second (2005), third (2006), fourth (2007), fifth (2008), sixth (2009) and seventh (2010) symposia, the *Eighth International Symposium on Recent Advances in Environmental Health Research (2011)* served as a platform to exchange innovative ideas and communicate the latest advances in scientific research and new developments on important environmental and human health topics including: Emerging Topics in Computational Biology and Environmental Modeling; Environmental Toxicology and Health Risk Assessment; Health Disparities and Environmental Security; Medical Geology and Human Health; Nanoscience, Nanotechnology and Nanotoxicology, Natural Resources Damage Assessment and Management; and New Frontiers in Environmental Health Research [1].

The symposium attracted 304 participants from 22 countries representing all five continents. A total of 196 scientific presentations were made across the disciplines of environmental health, biomedical and clinical sciences, and public health. The scientific program included seven plenary sessions where oral/plenary presentations were given by forty six invited speakers. In addition, there were two poster sessions—one for faculty and professional scientists, and one for students that included awards for best posters presentations at four levels of the educational pipeline including high school, undergraduate, master and doctorate levels. Three certificates and prizes (first, second and third) were awarded for each education level.

Original contributions were solicited on relevant topics of the Symposium. As in the past, authors were asked to access the journal’s website and submit their full length manuscripts. Submitted manuscripts were processed and sent out for peer-review by environmental and public health experts in their respective fields. A rigorous peer-review process was conducted as previously described [1], and according to the high publication standard of International Journal of Environmental Research and Public Health [3].

I wish to extend special thanks to Dr. Lucio Miele (Director of the Cancer Institute and Ergon Professor of Medicine and Pharmacology at the University of Mississippi Medical Center) for serving as Banquet Speaker. Dr. Miele presented an excellent lecture on the cancer incidence and mortality in Mississippi. He provided valuable information on cancer epidemiology in Mississippi, with a special emphasis on the environmental causes of cancer and genetic risk factors for specific cancer subtypes. Special thanks are also extended to Dr. Gilbert S. Omenn (Professor of Internal Medicine, human genetics and public health, and Director of the Center for Computational Medicine and Biology at the University of Michigan), and Dr. Perti J. Hakkinen (Senior Toxicologist, and Toxicology and Environmental Health Science Adviser at the National Library of Medicine) for serving as Distinguished Speakers for the Biomedical Sciences and Health Information Lecture Series that is held in conjunction with the Symposium. Dr. Omenn presented on the development of omics-based profiling tests for toxicology and clinical trials, and introduced the emerging Human Proteome Project which holds potential to help fill the gap between genome and phenome. Dr. Hakkinen presented on important information resources available at the National Library of Medicine for research and education on health risk assessment and management of environmental compounds. Other plenary presentations and keynote addresses were made by prominent biomedical and environmental health scientists and engineers with research expertise in cancer, diabetes, HIV/AIDS, infectious and parasitic diseases, cardiovascular diseases, neurodegenerative diseases, gene-environment interactions, nanoscience and nanomedicine, emerging technologies, health disparities and other environmentally-related illnesses. These important health issues were associated with the symposium topics [1,2].

Session chairpersons included Dr. Aage Haugen, National Institute of Occupational Health, Oslo, Norway; Dr. Stella Anyangwe, World Health Organization Country Office, Pretoria, South Africa; Dr. Mario Azevedo, Jackson State University, College of Public Service, Jackson, Mississippi, USA; Dr. Gloria Calaf, University of Tarapaca, Arica, Chile; Dr. Edmond Creppy, University of Bordeaux, Faculty of Pharmacy, Bordeaux, France; Dr. Jimmy Efird, Brody School of Medicine, Center for Health Disparities Research, Greenville, North Carolina, USA; Dr. Joseph Landolph, University of Southern California, School of Pharmacy, Los Angeles, California, USA; Dr. James Maddirala, Jackson State University, Jackson, Mississippi, USA; Dr. Dora N. Mbanya, University of Yaounde, Faculty of Medicine, Yaounde, Cameroon; Dr. Loretta Moore, Jackson State University, School of Engineering, Jackson, Mississippi, USA; Dr. Marinelle Payton, Jackson State University, School of Health Science, Jackson, Mississippi, USA; Dr. James Perkins, Jackson State University, RTRN-Data Technology Coordinating Center, Jackson, Mississippi, USA; Dr. Hector Rubio-Arias, Autonomous University of Chihuahua, College of Science, Chihuahua, Mexico; Dr. Karam Soliman, Florida A&M University, College of Pharmacy, Tallahassee, Florida, USA; Dr. Maria Torres, University of the Republic, Faculty of Chemistry, Montevideo, Uruguay; Dr. Robert Whalin, Jackson State University, School of Science and Technology, Jackson, Mississippi, USA; Dr. William M. Southerland, Howard University, College of Medicine, Washington DC, USA; and Dr. Hongtao Yu, Jackson State University, School of Science and Technology, Jackson, Mississippi, USA.

I would like to commend the authors for their involvement and cooperation, and for their outstanding contributions to advancing scientific research and facilitating informed decision making in the critical area of environmental sustainability and public health protection. Special thanks are also extended to all the peer-reviewers who took time from their busy schedules to carefully and critically review each of the manuscripts. Special appreciations are also extended to all my colleagues and staff who worked very hard to make the symposium a total success.

Special thanks are extended to Dr. Carolyn W. Meyers (President), Dr. Mark G. Hardy (Provost & Vice President for Academic Affairs), Dr. Felix Okojie (Vice President for Research and Federal Relations), and Dr. Mary Myles (Director of Title III Program) for their administrative support. On behalf of the entire organizing committee, the greatest acknowledgments go to our major symposium sponsors including the National Institutes of Health (NIH) RCMI-Center for Environmental Health, the U.S. Department of Education Title III Graduate Education Program, the U.S. Environmental Protection Agency, the JSU Office of Academic Affairs, and the JSU Office of Research and Federal Relations.

Special thanks are also extended to Mrs. Rose Foster and Mrs. Wilma Templin-Branner from Oak Ridge Institute for Science and Education, and Drs. Kenneth Ndebele and Barbara Graham from Jackson State University, for their continued support and help with the organization of the pre-symposium workshop on the *National Library of Medicine Web-Based Resources for Environmental Health and Biomedical Research.* Training participants on how to access and retrieve important environmental health and biomedical research information from relevant web-based resources was the major emphasis of the workshop.

The next (9th) Symposium will be held at the Jackson Marriott Hotel, from September 16–19, 2012. I invite all past Symposium participants, as well as new biomedical and environmental health scientists and engineers who are interested in solving global environmental and public health challenges, at the next meeting.
